# Complex Recanalization of Chronic Total Occluison Supported by Minimal Extracorporeal Circulation in a Patient with an Aortic Valve Bioprothesis in Extraanatomic Position

**DOI:** 10.1155/2018/4975412

**Published:** 2018-04-05

**Authors:** Ruben Jansen, Brigitte Bathgate, Alexander Bufe, Jan-Erik Guelker

**Affiliations:** ^1^Department of Cardiology, Heartcentre Niederrhein, Helios Clinic Krefeld, Krefeld, Germany; ^2^Institute for Heart and Circulation Research, University of Cologne, Cologne, Germany; ^3^University of Witten/Herdecke, Witten, Germany

## Abstract

Percutaneous coronary intervention (PCI) of chronic total occlusion (CTO) still remains a major challenge in interventional cardiology. This case describes a complex PCI of the left main coronary artery and of a CTO of the right coronary artery using a minimal extracorporeal circulation system (MECC) in a patient with an aortic valve bioprothesis in extraanatomic position. It illustrates that complex recanalization strategies can be solved combining it with mechanical circulatory support technologies.

## 1. Introduction

Recanalization of chronic total occlusion (CTO) remains a complex procedure in interventional cardiology. CTO of a coronary artery can be identified in up to 20% among patients with a clinical indication for coronary angiography. With the advent of novel recanalization techniques and emerging devices, percutaneous coronary interventions (PCIs) have become a promising treatment option for these patients [1–5].

The minimal extracorporeal circulation (MECC) system (Maquet, Hirrlingen, Germany) is a preconnected closed system with a centrifugal pump and a membrane oxygenator. It is a tip-to-tip heparin coated system which needs less than 500 ml priming volume. Venous blood returns through active drainage with a centrifugal pump instead of a roller pump to a membrane diffusion oxygenator. Venous reservoir and cardiotomy suction of shed blood is not necessary [6]. We report a case of complex coronary recanalizations using the MECC support in a patient with a calcified coronary artery disease.

## 2. Case Report

We present a case of a 66-year-old woman with advanced coronary artery disease (CAD). In 2013, she underwent coronary artery bypass graft (CABG) surgery with an IMA bypass to the left anterior descending (LAD) and implantation of an aortic valve bioprothesis in extraanatomic position with a conduit from the apex of the left ventricle to the descending aorta for treatment of a symptomatic aortic valve stenosis. This procedure was chosen as a “porcelain” aorta did not allow an implantation in a regular position.

Due to recurrent angina pectoris (CCS III) and dyspnoe (NYHA III), a coronary angiography was performed exhibiting an occlusion of the IMA bypass graft, a high-grade stenosis of the left main coronary artery (LCA) and a chronic total occlusion (CTO) of the right coronary artery (RCA).

**Figure 1:**
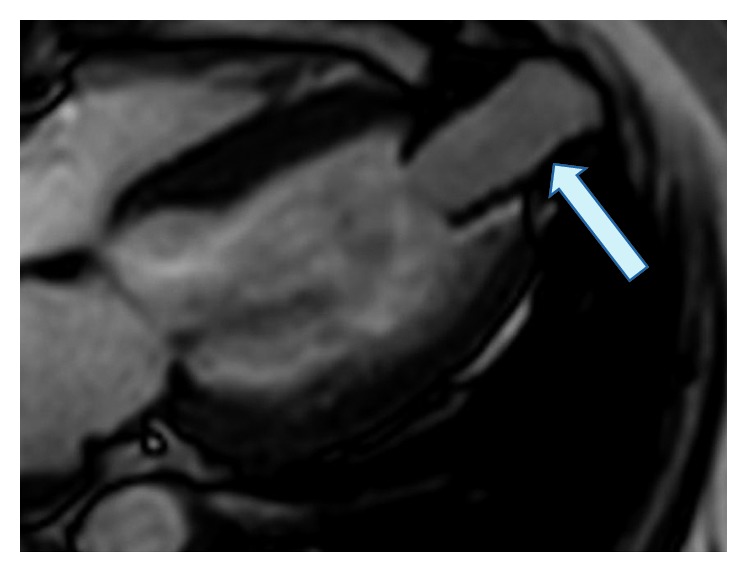
MRI shows a conduit from the apex of the left ventricle (arrow).

**Figure 2:**
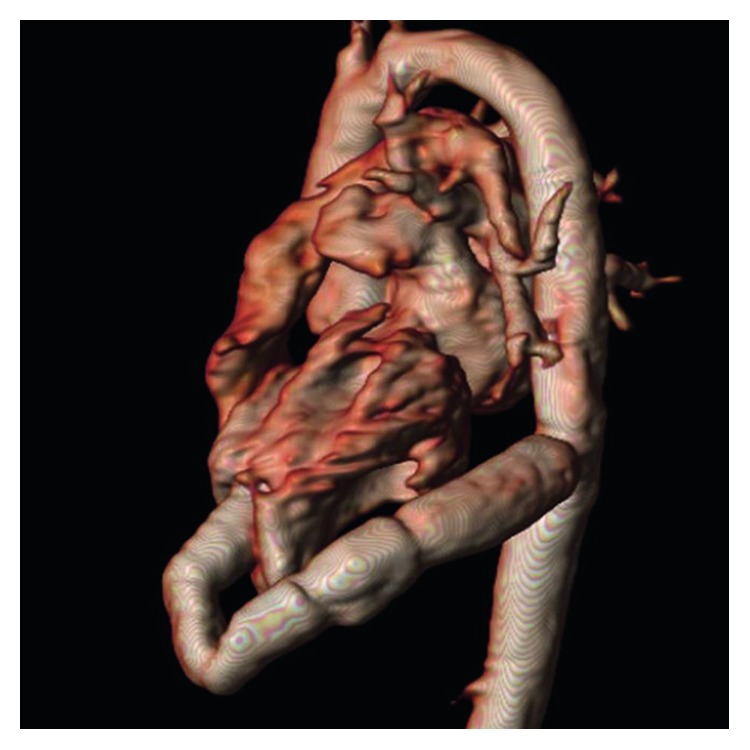
MRI shows a conduit from the apex of the left ventricle to the descending aorta.

Transoesophageal and transthoracal echocardiography revealed a reduced left ventricular function with an ejection fraction of 45% with mild hypokinesia of the inferior wall. The function of the bioprothesis was regular. This was also confirmed by magnet resonance imaging (MRI) of the heart. The prothesis was positioned 8 cm distal in the conduit (Figures 1 and 2).

The local heart team decided to perform a percutaneous coronary intervention (PCI) of the left main coronary artery and a PCI of the RCA-CTO assisted by a MECC system.

Before starting the procedure, anesthesia and mechanical ventilation were installed. To prevent thrombembolic complications, heparin was given during the interventions guided by the activated clotting time (>300 sec). To introduce the minimal extracorporeal circulation system, an arterial 14 French and a venous 21 French access were established. Following a loading dose of 600 mg clopidogrel, a 7F JL4 guiding catheter was advanced to the left main coronary artery, and 0.014” floppy guide wires were introduced into the LAD, left circumflex artery (LCX), and ramus intermedius.

**Figure 3:**
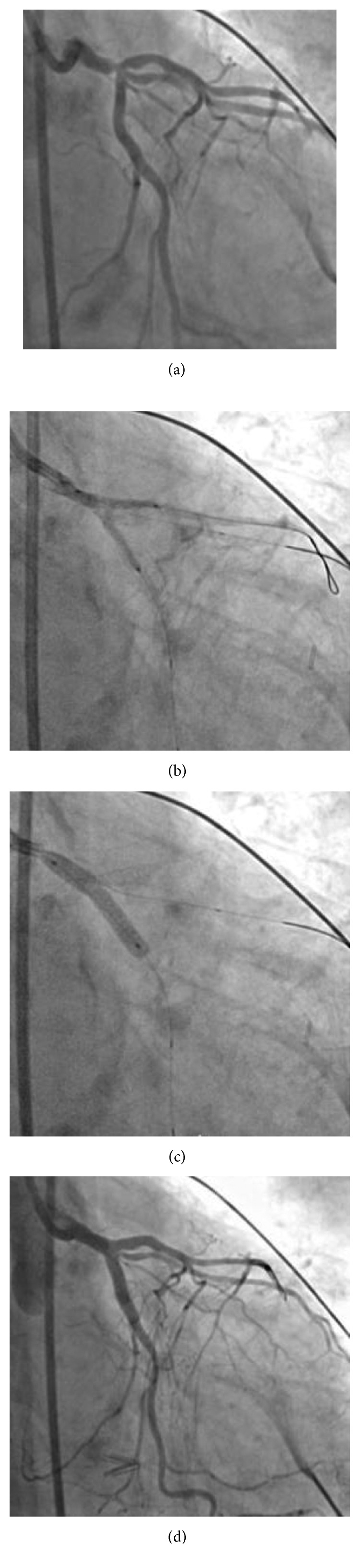
Complex recanalization of the left main coronary artery (a–d).

The left main coronary artery was stented in the direction of the LAD and LCX using the mini-crush technique; a 2.75 × 15 mm and a 3.5 × 23 mm drug-eluting stent (DES) were deployed at 18 and 20 atmospheres, respectively (Figure 3).

**Figure 4:**
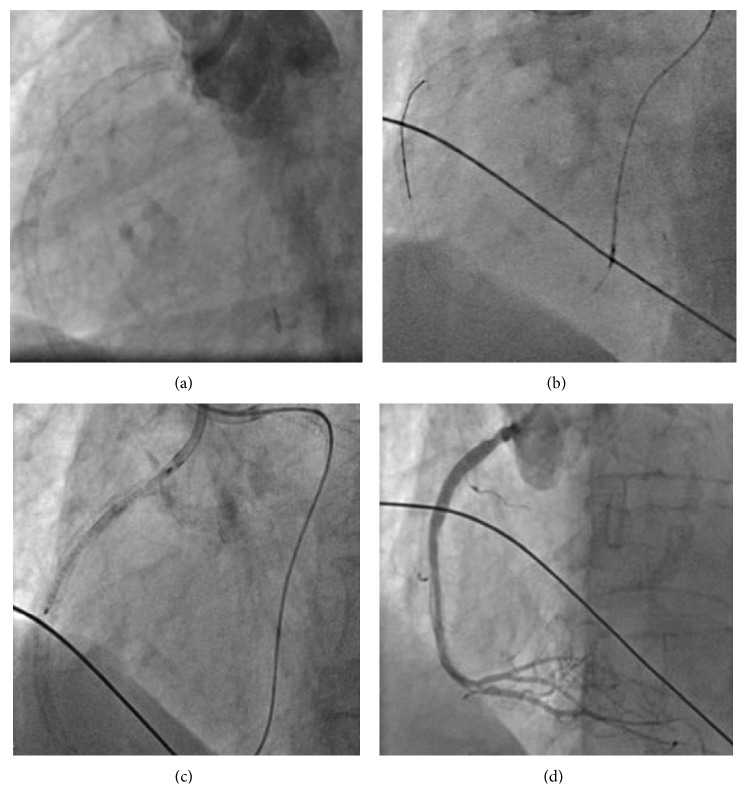
Retrograde recanalization of the RCA (a–d).

After two failed antegrade attempts in the past, the retrograde CTO technique was applied to recanalize the RCA. For recanalization, a Sion wire (Asahi Intecc, Japan) and a Corsair microcatheter (Asahi Intecc, Japan) were used. Wire and microcatheter passed the occluded segment, and after multiple balloon dilatations, a 3.5 × 38 mm DES was implanted (Figure 4). After the procedure, we checked the result with intravascular ultrasound (IVUS). A dual antiplatelet therapy consisting of 100 mg of aspirin once daily and 75 mg of clopidogrel daily for at least 6 months was continued. Artificial ventilation was stopped on the same day, and the patient could leave the hospital five days later.

## 3. Discussion

The MECC system has been introduced in clinical practice in 1999. Initially, it was applied in aortic valve surgery as well as in other cardiac surgical procedures [7, 8].

Complex interventional procedures like PCI of the left main or of the CTO often require a hemodynamic support. The advantages of the MECC system in comparison to other mechanical circulatory support technologies like the intra-aortic balloon pump (IABP) or the left ventricular assist device (LVAD) are an immediate hemodynamic stabilization and additional support by unloading the right ventricle [9]. As a potential alternative, the Impella CP microaxial pump (Abiomed, USA) has been introduced. Several trials have shown before that cardiac survival was higher in patients with complete revascularization when compared to patients with incomplete revascularization [10, 11].

Our case demonstrates that by combining advanced recanalization techniques and the MECC system complex, coronary conditions which are so far considered as intractable by interventional means can be treated successfully. The combination of both, high-standard CTO-PCI and an extracorporeal circulation support system, allows us to pursue the interventional procedure. A profound knowledge and a confident command of CTO techniques are required for this kind of intervention. Nevertheless, a strong cooperation between interventional cardiologists and cardiac surgeons is still necessary as part of the heart team [12].
